# Art of prevention: Our approach to the measles-mumps-rubella vaccine in adult patients vaccinated against measles before 1968 on biologic therapy for the treatment of psoriasis^☆^

**DOI:** 10.1016/j.ijwd.2019.11.003

**Published:** 2019-12-27

**Authors:** Audrey Worth, Reid A. Waldman, Kevin Dieckhaus, Marti J. Rothe

**Affiliations:** a Department of Arts & Sciences, University of Connecticut, Storrs, Connecticut; b Department of Dermatology, University of Connecticut, Farmington, Connecticut; c Division of Infectious Diseases, University of Connecticut, Farmington, Connecticut

**Keywords:** Measles, MMR, CDC guidelines, Psoriasis, Biologic agents, Vaccines

## Abstract

**Background:**

In response to the evolving measles epidemic in the United States, the Centers for Disease Control and Prevention recommended that some adults be revaccinated against measles because they may have inadequate immunity against the virus. Patients receiving biologic medications for psoriasis face a clinical dilemma because they may be at an increased risk of developing severe measles; however, vaccination with the measles-mumps-rubella (MMR) vaccine is not recommended for those on biologic therapy according to the American Academy of Dermatology-National Psoriasis Foundation guidelines.

**Objectives:**

This study aimed to review available research on the safety and efficacy of live-attenuated vaccines in individuals receiving biologic therapy for psoriasis and to discuss our approach to vaccinating individuals on biologic agents for psoriasis with the MMR vaccine.

**Methods:**

A review of the literature was performed via PubMed search. Our institution’s anecdotal experiences are also discussed.

**Results:**

Data, although limited, are available suggesting that live-attenuated vaccines may be safe for individuals on tumor necrosis factor-alpha inhibitors for psoriasis. Inadequate data are available for patients receiving other biologic medications.

**Conclusion:**

Providers should engage in shared decision-making to determine whether patients on tumor necrosis factor-alpha inhibitors for psoriasis should receive the MMR vaccine without an interruption in biologic therapy.

## Introduction

In response to the recent measles epidemic in the United States, the Centers for Disease Control and Prevention (CDC) has recommended that individuals born after 1957 and vaccinated for measles before 1968 be revaccinated with the measles-mumps-rubella (MMR) live-attenuated vaccine (CDC, 2019). The CDC issued this recommendation because the measles vaccine administered prior to 1968 was an inactivated vaccine that may not provide lifelong protection to recipients. This recommendation makes the MMR vaccine the only live-attenuated vaccine that is routinely recommended for adults outside of those recommended prior to international travel.

This information is relevant to dermatologists for two reasons: 1) Patients receiving certain biologic medications may be at an increased risk of experiencing significant morbidity and even mortality from a measles infection, and 2) the current American Academy of Dermatology-National Psoriasis Foundation (AAD-NPF) joint psoriasis guidelines recommend against the administration of live-attenuated vaccines to individuals receiving biologic medications without an interruption in biologic therapy, making administration of the MMR vaccine challenging in this population (Menter et al., 2019, Rafat et al., 2013).

This article reviews the available literature regarding the safety and efficacy of the MMR vaccine in patients receiving biologic medications and details our approach to the administration of the MMR vaccine to patients with psoriasis on biologic medications.

## Risk of measles-related complications in individuals on biologic medications

The risk of severe measles in patients with psoriasis on biologic medications is unknown. There are no published cases of measles in patients on biologic therapy for psoriasis because prior to the recent measles epidemic, measles was eradicated in the United States in 2000 (i.e., prior to the introduction of biologic medications for psoriasis). Although there are also no cases recorded of measles in any individual receiving an IL-17 inhibitor, an IL-12/23 inhibitor, or an IL-23 inhibitor, but a report exists of measles in a patient with rheumatoid arthritis on etanercept, a tumor necrosis factor (TNF) alpha inhibitor, that resulted in severe leukopenia and hospitalization (Takahashi et al., 2010). Based on this report and on the observation that individuals on biologic medications for psoriasis are at an increased risk of developing viral upper respiratory tract infections, it is plausible to assume that patients with psoriasis receiving biologic medications may be at an increased risk of developing severe measles and therefore may benefit from the measles revaccination strategy recommended by the CDC.

## Safety of MMR vaccine in individuals on biologic medications

Unfortunately, vaccinating individuals on biologics for psoriasis against measles in accordance with CDC guidelines is challenging because there is a paucity of data on the safety and efficacy of the MMR vaccine in this population. Because of the limited data on this topic and concerns that immunosuppressed individuals are at increased risk of experiencing a severe adverse event (e.g., vaccine-related infection) from administration of live-attenuated vaccines, the AAD-NPF guidelines conservatively recommend “discontinuation of all biologic agents” prior to administration of a live vaccine (Menter et al., 2019).

However, the guidelines acknowledge that there are some reports supporting the safety of administering live-attenuated vaccines to individuals on biologic medications. Of note, the MMR vaccine is administered routinely to other immunosuppressed populations, including those with HIV infection (children age <5 years with CD4% >15% or older individuals with absolute CD4+ count >200 cells/mL) and allogeneic bone marrow transplant recipients (>2 years after transplant with no evidence of graft-versus-host disease and not currently receiving immunosuppressive agents; McLean et al., 2013, Tomblyn et al., 2009).

We identified two studies that evaluated the safety of live vaccines in patients on biologics. Zhang et al. (2012) conducted a retrospective Medicare claims database review of the safety of the live-attenuated herpes zoster vaccine in immunosuppressed individuals. This study identified 551 patients on TNF-alpha inhibitors who had received the live-attenuated herpes zoster vaccine while on anti-TNF therapy. None of the patients developed a vaccine-related infection. Huber et al. (2018) conducted a retrospective analysis of the safety of live-attenuated vaccines administered to patients on immunosuppressive and biologic medications who attended Swiss travel clinics between 2008 and 2015. This study identified two patients on adalimumab who received the MMR vaccine. Neither patient developed a severe vaccine-related adverse event. This study also identified six additional patients on TNF-alpha inhibitors who had received either the yellow fever or oral typhoid vaccine (i.e., live-attenuated vaccines) and one patient on ustekinumab (IL12/23 inhibitor) who had received the yellow fever vaccine. None of these patients developed a severe vaccine-related adverse event either. The indication for which the aforementioned patients were receiving biologic medications was not reported.

## Efficacy of MMR vaccine in individuals on biologic medications

Unfortunately, although there are favorable, albeit somewhat limited, safety data on the MMR vaccine in individuals on biologic medications, there are no studies evaluating the immunogenicity of the MMR vaccine in this population (i.e., whether individuals on biologic medications respond to the MMR vaccine). However, in vivo immunogenicity studies support that live-attenuated vaccines can be effective in individuals on biologic medications, including TNF-alpha inhibitors, although none of these studies specifically evaluated immunogenicity of the MMR vaccine (Schleker et al., 2018).

## Our approach

We offer patients with psoriasis who are on biologic medications the MMR vaccine if indicated by CDC guidelines. We do not routinely refer patients to their primary care physician or to an infectious disease specialist for clearance to receive the MMR vaccine because we have previously discussed this topic in detail with our university’s infectious disease specialists (K.D. is the division chief of Infectious Disease at the University of Connecticut). However, referring patients with complex comorbidities and/or other possible sources of immunosuppression to an infectious disease specialist is reasonable.

We also do not routinely obtain MMR titers prior to vaccination because this is not recommended by the CDC. However, routinely checking MMR titers and only vaccinating nonimmune patients would not be unreasonable because this would decrease the likelihood that an individual on biologic therapy is unnecessarily revaccinated. If titers are obtained, they should be interpreted in the context of reference ranges provided by the laboratory for that specific laboratory test. Laboratories establish cutoffs below which patients are deemed nonimmune.

We explain to patients on TNF-alpha inhibitors that they may be at an increased risk of developing complications should they contract measles, owing to their biologic-related immunosuppression. In contradistinction, we explain to patients on non-TNF biologics that their risk of developing severe measles is unknown. We inform all patients considering receiving the MMR vaccine that current AAD-NPF guidelines recommend an interruption in therapy for vaccination but that some data support that vaccination while on TNF-alpha inhibitors and certain other biologic medications is safe (Menter et al., 2019). We explain that an interruption in TNF-alpha inhibitor therapy may cause patients to be less responsive to their biologic medication upon reinitiation after vaccination.^10^

After informing patients of the risks and benefits of receiving the MMR vaccine while on a TNF-alpha inhibitor, we allow patients to choose whether they want to temporarily discontinue their TNF-alpha inhibitor for vaccination. We counsel patients who choose to remain on therapy while receiving the MMR vaccine about signs of vaccine-related infection and counsel them to contact our office and seek care at their local emergency department should these signs and/or symptoms develop. We are able to obtain the MMR vaccine in our department because we are a university hospital-based practice. For providers who do not vaccinate at their offices, referral back to primary care with a letter endorsing vaccination is reasonable.

## In the event of a local measles outbreak

In the event of a local measles outbreak, local health departments will provide special guidance and recommendations about vaccination of “groups at increased risk for measles because of a measles outbreak” (CDC, 2019) For example, in response to the recent measles outbreak in New York, the New York City Health Department issued special recommendations to “administer an early, extra dose of MMR to infants aged 6 to 11 months” who were residing or frequently visiting areas of the outbreak (New York City Health, 2019). This specific recommendation was issued because this age group was deemed to be at high risk based on the epidemiology of the measles outbreak in New York City.

## Conclusion

As the incidence of measles increases in the United States, patients with psoriasis who are on biologic medications may be at an increased risk of developing severe measles. This risk is most apparent in individuals vaccinated against measles prior to 1968 who may no longer have protective immunity against the virus. Although there is undoubtedly a need for additional research about the safety and immunogenicity of the MMR vaccine in patients receiving biologic medications for psoriasis, dermatologists need to address patient immunity to measles in the interim because this poses an increasing threat.

In our opinion, all patients with psoriasis who are on biologic medications and require the MMR vaccine per CDC guidelines should be offered the vaccine. This is especially true for patients who reside in or frequently visit areas of measles resurgence. Additionally, shared decision-making should be used to determine whether a patient should interrupt biologic therapy for vaccination, as recommended by the AAD-NPF guidelines.

## Conflict of Interest

None

## Funding

None.

## Study Approval

The authors confirm that any aspect of the work covered in this manuscript that has involved human patients has been conducted with the ethical approval of all relevant bodies.
